# Supplementary material for: Evaluation of Nitrogen and Carbon Stable Isotopes in Filter Feeding Bivalves and Surficial Sediment for Assessing Aquatic Condition in Lakes and Estuaries
Source: Water (Basel). Author manuscript; Available in PMC 2023 Nov 16. (PMC9888023; doi:10.3390/w14223712)
Supplement: Supplement1 — Table S1: Location data for study lakes and watershed size (ha) for lakes and estuaries; Table S2: Number, size data, δ15N and δC13 for Mussels (UN) from 26 Lakes; Table S3: Number, size data, δ15N and δC13 and sample site locations for Mercenaria mercenaria from a total of Estuaries 28 stations from nine Estuaries. [file NIHMS1863475-supplement-Supplement1.zip › water-1984815-supplementary.pdf]

water

MDPI

Supplementary materials

Evaluation of Nitrogen and Carbon Stable Isotopes in Filter Feeding Bivalves and Surficial Sediment for Assessing Aquatic Condition in Lakes and Estuaries

James L. Lake [1,*], Jonathan R. Serbst [1], Anne Kuhn [1], Michael Charpentier [2] and Nathan J. Smucker [3]

[1] US Environmental Protection Agency, Office of Research and Development, 27 Tarzwell Drive, Narragansett, RI, 02882, USA
[2] General Dynamics Information Technology, 27 Tarzwell Drive, Narragansett, RI, 02882, USA
[3] US Environmental Protection Agency, Office of Research and Development, 26 Martin Luther King Drive W., Cincinnati, OH, 45268, USA
* Correspondence: lake.jim@epa.gov

Table S1. Location data for study lakes and watershed size (ha) for lakes and estuaries.

Lake Name	Lake Lat.	Lake Long.	Wastershed size (ha) with water removed
**51 Lakes**			
Alton	41.443162	-71.718536	22570
Asa	41.459212	-71.513120	310
Ashville	41.500854	-71.756127	70
Belleville	41.563214	-71.480517	910
Boone	41.583003	-71.673801	570
Bowdish	41.923120	-71.774661	650
Breakheart	41.598387	-71.701314	1590
Browning Mill	41.558071	-71.689525	1410
Burlingame	41.909663	-71.745370	480
Carbuncle	41.697874	-71.773378	450
Chapman	41.380264	-71.794961	1840
Deep	41.391267	-71.662400	20
Eisenhower	41.620902	-71.716280	840
GilbertS	41.635722	-71.556017	1230
Gorton	41.705270	-71.458830	300
Hundred	41.500151	-71.542055	2360
Indian	41.480009	-71.468058	210
Jamestown	41.531310	-71.372326	80
JL Curran	41.752465	-71.549023	210
Keech	41.879793	-71.687589	1540
Larkin	41.469520	-71.558576	30
Little	41.705795	-71.406844	50
Locustville	41.516659	-71.721313	3270
Lower Beach	41.571233	-71.784443	160
Meadowbrook	41.440659	-71.690722	1590
Mishnock	41.653108	-71.588596	60
Nonquint	41.564006	-71.195182	1710
OakSwamp	41.827830	-71.540068	180
Pasquisett	41.426073	-71.631182	1190
Quidnick	41.679780	-71.678483	560
Schoolhouse	41.400973	-71.668023	410
Secret	41.554608	-71.478145	620
Simmons Mill	41.539929	-71.148572	380
Spring Green	41.735011	-71.409398	160
Stafford	41.640217	-71.156126	400
Tarbox	41.635157	-71.569088	410
Tiogue	41.678282	-71.550350	630
Tucker	41.422160	-71.551828	230
Turner	41.837815	-71.339272	13640
Upper Dam	41.705572	-71.552801	80

UpperSlatersville	41.989944	-71.596890	5030
Wakefield	41.963128	-71.792426	390
Wallum	42.001032	-71.765288	490
Warwick	41.723752	-71.412675	380
Watchaug	41.384054	-71.691207	1660
Waterman	41.876649	-71.586161	1980
Wilson	41.969313	-71.726221	2870
Wincheck	41.515939	-71.771544	1030
Worden's	41.439087	-71.574914	6210
Wyoming	41.519497	-71.697449	15000
Yawgoo	41.511071	-71.573107	400
Nine Estuaries [1]			
Bass River			3830
Great Island			7320
Great Pond			4960
Little Pond			620
Mill Creek			1100
Narragansett Bay (RI + MA)			275000
Ninigret Pond			3650
Sage Lot Pond			250
Three Bays			6000

[1] Sample site locations for estuaries are in Supplemental Table 3

Table S2. Number, size data, δ^{15}N and δC^{13} for Mussels (UN) from 26 Lakes.

Lake	Number collected	Mean length mm	Minimum Length mm	Maximum Length mm	Mean δ^{15}N$_{UN}$ ‰	Mean δ^{13}C$_{UN}$‰
Alton	6	70	53	83	8.2	-32.8
Belleville	6	89	64	110	9.3	-30.9
Browning Mill	4	86	78	94	6	-32.4
Chapman	2	75	72	78	9.4	-31.3
Eisenhower	10	85	65	100	4.5	-31.5
Gorton	4	84	78	92	12.1	-28.6
Hundred Acre	4	83	78	90	7.6	-33.3
Larkin	4	74	70	75	7.7	-26.6
Little	6	75	58	85	8.3	-26
Locustville	4	65	56	72	7	-33.2
Meadowbrook	6	71	56	89	7.5	-30.8
Mishnock	5	79	67	84	9.1	-32.3
Nonquint	9	68	60	87	8.4	-28
OakSwamp	5	69	55	80	7.8	-28.7
Pasquisett	3	54	49	61	5.8	-32.9
Quidnick	6	81	78	86	5.3	-30.5
School House	4	60	50	68	3.4	-29.1
Stafford	4	71	56	90	7	-24.3
Tiogue	4	75	68	81	12.2	-26.7
Tucker	12	66	50	85	3.8	-29.3
Turner	2	90	90	90	15.5	-29.9
Upper Dam	4	73	60	86	11.6	-29.9
Upper Slatersville	20	73	63	87	9.6	-31.8
Wordens	5	51	47	56	6.8	-30.5
Wyoming	5	91	75	100	8	-31.6
Yawgoo	4	68	66	69	5.5	-28.9

Table S3. Number, size data, $\delta^{15}N$ and δC^{13} and sample site locations for Mercenaria mercenaria from a total of Estuaries 28 stations from nine Estuaries.

Estuary	Sample Site	Number Collected	Minimum length mm	Maximum length mm	Mean length mm	Mean $\delta^{15}N_{MM}$ ‰	Mean $\delta^{13}C_{MM}$‰	Site LAT	Site LON
BassRiver	Freydis	3	56	87	74	9.8	-18.2	41.699613	-70.166836
BassRiver	Thorwold	4	46	95	75	9.6	-18.1	41.700126	-70.167413
BassRiver	LEricson	4	46	108	77	9.7	-18	41.700682	-70.167941
BassRiver	Norseman	4	64	94	84	9.3	-17.6	41.701101	-70.168411
GreatPond	GPmassa	5	41	65	54	9.9	-17.6	41.550954	-70.585121
GreatPond	GPhiawa	6	42	55	51	10	-17.7	41.553172	-70.585372
GreatPond	GPcypress	4	57	68	63	10	-17.8	41.559225	-70.585828
LittlePond	LPoutlet	2	57	60	59	11.9	-17.2	41.54678	-70.788767
LittlePond	LPmassa	4	56	88	71	11.9	-17.1	41.550561	-70.588091
LittlePond	LPhiawa	3	60	80	73	12.2	-17.3	41.552684	-70.589049
MillCreek	MCsite 1	2	82	82	82	13.4	-19.1	41.585727	-71.449188
NarrBay	FtGetty	6	46	65	55	13	-17.4	41.700126	-70.167413
Ninigret	Nini1	4	45	64	54	7.4	-17.8	41.34572	-71.686952
Ninigret	Nini2	4	65	83	72	8.3	-18.1	41.34667	-71.684967
Ninigret	Nini3	4	38	80	56	7.5	-18.8	41.347637	-71.681362
PtJudithPond	GalBoatRamp	1	52	52	52	10.6	-18.3	41.383207	-71.507196
PtJudithPond	BasinDrive	6	37	68	52	11.5	-18.5	41.385583	-71.509218
PtJudithPond	EShoreDr	3	39	55	47	11.7	-17.6	41.389817	-71.499707
PtJudithPond	FranksNeck	1	85	85	85	10.7	-18.5	41.393099	-71.498307
SageLot	sage1	4	52	80	64	7.9	-18.4	41.551785	-70.509256
SageLot	sage2	3	62	80	69	7.5	-18.8	41.552302	-70.507121
SageLot	sage3	5	50	78	63	7	-18.7	41.552484	-70.507857
ThreeBays	Seaview	2	60	65	63	9.6	-19.6	41.609017	-70.39992
ThreeBays	Cross	1	73	73	73	9.2	-19.6	41.609327	-70.434881
ThreeBays	CotuitTD	1	68	68	68	9.3	-18.5	41.616451	-70.43343
ThreeBays	Ropes	2	60	62	61	9.2	-19.7	41.62049	-70.433126
ThreeBays	BridgeSt	2	64	67	66	9.2	-18.3	41.622095	-70.393327
ThreeBays	BaySt	5	41	84	62	9.3	-18.7	41.628009	-70.395847
